# Health Tract, No. 236

**Published:** 1865

**Authors:** 


### "EVER THANKFUL."

A BEAUTIFUL theme, and admirably was it handled in a public discourse a few years ago by the Rev. Mr. Scovell, of Springfield, Ohio; and no wonder that his generous and affectionate people called for its publication. Great reason is there that we should be "ever thankful," thankful and humble too!

"Ten thousand times His goodness seen
Ten thousand times that goodness grieved."

"Thankful," as the good and loved and revered Dr. James W. Alexander used often to say in the opening Sabbath morning prayer, "for the proper use of our reason." Thankful that we live in a land where the glorious privilege is awarded to all of worshiping God according to the dictates of their own consciences, none being near "to make afraid." Thankful for being permitted to live under the freest and most glorious government on the face of the globe; glorious in its productiveness, glorious in the extent of its territory, in the beauty, variety, and healthfulness of its climate; for its churches, its free schools; and glorious as a power to be feared and respected among the nations of the earth.

Thankful am I for house, and home, and safe abode; thankful that all my children are spared to me in affection and lovingness, and uninterrupted good health for another year; renewedly thankful every morning to hear the quick patter of their little feet, from bed to fireside, overhead, being the first telegram of the morning that another night has been passed in safety, and that they have awaked again to health and joyousness; thankful ever and as often as we meet around the breakfast-table, without a cloud upon their brows, and not a murmur from their lips; thankful for the good warm breakfast of a winter's morning; thankful for the appetite to eat it, and thankful truly that there is no unpleasant reminder thereafter, telling of dyspepsia and its thousand horrors; and not less thankful, in the after part of the day, that they have returned from school without disaster or accident, and though looking weary enough for the exercise of six hours' study, which the inconceivable stupidity of school directors continues to impose on minds just budding into strength, yet soon waking up, in the prospect of a good dinner, an afternoon's play, and music, and laughter, and mirth around the blazing fire in the parlor, while the howling of the winter wind through the lattice, and the beating of the sleet on the pane, and the street lamps glimmering in their loneliness through the pitchy darkness, by contrast increase the comfort and add to the genial joy; thankful for the privilege of living as a candidate, not for the "White House," but for one of those mansions in the skies, which the Lord Redeemer has gone before to prepare for all who live and die faithful to him, his honor and his cause. Reader! it is worth while to cultivate a thankful disposition. It mitigates life's sorrows, it intensifies every gladness, it sweetens sleep, invigorates digestion, and tonifies the whole man. Both body and soul are benefited largely and always by the cultivation of a thankful nature, and its sweet influences spread around from heart to heart, making, in truth, oftentimes, a young heaven here below; and for the new year, so soon to dawn upon us, let us all resolve, in reference to that Eye which, in kindness to us, "never sleeps," to be

"EVER THANKFUL."

